# The need for strengthening the influenza virus detection ability of hospital clinical laboratories: an investigation of the 2009 pandemic

**DOI:** 10.1038/srep43433

**Published:** 2017-03-10

**Authors:** Shigui Yang, Yuqing Zhou, Yuanxia Cui, Cheng Ding, Jie Wu, Min Deng, Chencheng Wang, Xiaoqing Lu, Xiaoxiao Chen, Yiping Li, Dongyan Shi, Fenfang Mi, Lanjuan Li

**Affiliations:** 1 State Key Laboratory for Diagnosis and Treatment of Infectious Diseases, Collaborative Innovation Center for Diagnosis and Treatment of Infectious Diseases, The First Affiliated Hospital, College of Medicine, Zhejiang University, Hangzhou 310003, China; 2 Zhejiang Institute of Medical-care Information Technology, Hangzhou 311112, China; 3 Zhejiang Chinese Medical University, Hangzhou 310053, China

## Abstract

Most hospital clinical laboratories (HCLs) in China are unable to perform influenza virus detection. It remains unclear whether the influenza detection ability of HCLs influences the early identification and mortality rate of influenza. A total of 739 hospitalized patients with 2009 influenza A (H1N1) virus treated at 65 hospitals between May and December, 2009, in Zhejiang, China, were included based on identifications by HCLs and by public health laboratories (PHLs) of the Centers for Disease Control and Prevention. Of the patients, 407 (55.1%) were male, 17 died, resulting in an in-hospital mortality rate of 2.3%, and 297 patients were identified by HCLs and 442 by PHLs. The results indicated that a 24-hour delay in identification led to a 13% increase in the odds of death (OR = 1.13, P < 0.05). The time between onset and identification (3.9 days) of the HCL cohort was significantly shorter than that of the PHL cohort (4.8 days). The in-hospital mortality rate of the HCL group was significantly lower than that of the PHL group (1.0% vs. 3.2%, P < 0.05). HCL-based detection decreased the in-hospital mortality rate by 68.8%. HCL-based influenza virus detection facilitated early identification and reduced influenza mortality, and influenza detection ability of HCLs should be strengthened.

After pH1N1 virus was first identified in April 2009, it spread rapidly to almost all countries during 2009 and 2010^1^,^2^,^3^,^4^,^5^. As of March 2010, more than 17,700 deaths among laboratory-confirmed cases had been reported to the World Health Organization (WHO)^6^. The pH1N1 influenza epidemic, though it has ended, has raised significant concerns about how to reduce the incidence of complications in influenza patients and prevent them from developing critical cases of influenza and dying.

Global efforts to enhance early disease detection and increase diagnostic abilities have stimulated the formation of laboratory networks of influenza detection and treatment^7^. A variety of sophisticated detection methods of pH1N1 influenza, such as RT-PCR and rtRT-PCR, were recommended by the WHO^8^. However, most hospital clinical laboratories (HCLs) are still unable to carry out clinical influenza virus detection, and the majority of pH1N1 influenza patients were identified by public health laboratories (PHLs) based in institutes of the Centers for Disease Control and Prevention during the pandemic^9^.

Currently, it remains unclear whether the ability of HCLs to detect the influenza virus influences early case identification and antiviral therapy and in particular the mortality rate of influenza. During the pH1N1 influenza pandemic in 2009, we developed a cohort of 739 hospitalized patients whose infection was identified by different types of laboratories, which enabled us to assess the influence of the HCL influenza virus detection ability on early case identification and mortality rate.

## Results

### Demographic and clinical features of patients

Among the cohort of 739 hospitalized patients, 407 (55.1%) were male and 332 (44.9%) female, and 194 (26.3%) were children (<14 years old), 486 (65.9%) were aged 14–60, and 57 (7.7%) were more than 60 years old. Forty-seven (7.0%) had a body mass index higher than 30, and 43 (6.0%) were pregnant. In the cohort, 619 patients had a fever, and 578 had a cough, accounting for 98.3% and 96.5% of the patients, respectively. Among the 739 hospitalized patients, 146 (19.8%) were classified as critical and 17 died, resulting in an in-hospital mortality rate of 2.3%. Among the cohort of 739 hospitalized patients, 297 patients were identified by HCLs and 442 by PHLs. The proportion of metabolic disease in cases detected by PHLs was modestly higher than that in cases detected by HCLs (7.9% VS 4.4%), but the difference is not significant (P = 0.056) (Table 1). The symptoms of cough, white sputum and fatigue were the longest-lasting in the course of the disease, at 10.1 ± 6.1 days, 8.4 ± 5.2 days, and 7.1 ± 4.5 days, respectively. Cardiovascular diseases, pulmonary diseases, and allergy were the main comorbidities, and the numbers of patients with these three symptoms were 85 (12.0%), 62 (8.8%) and 58 (8.4%), respectively. The numbers of patients with pneumonia, acute liver injury and acute respiratory distress syndrome were 444 (70.9%), 127 (20.2%) and 87 (13.9%), respectively. (See Supplementary Table S1).

### Frequency and time intervals of the primary medical activities of patients with different outcomes

For the patients classified as critical, the average time intervals from symptom onset to identification, first hospital visit to identification, and symptom onset to the initiation of antiviral therapy with oseltamivir was 5.5 ± 4.1 days, 4.0 ± 3.3 days and 5.8 ± 4.5 days, respectively, significantly longer than those for the non-critical patients (4.4 ± 3.1 days, 2.8 ± 3.6 days and 4.3 ± 2.9 days, respectively). The frequency of hospital visits (1.9 ± 1.0 times) for the patients with critical cases was significantly higher than that (1.6 ± 1.1 times) for the patients with non-critical cases. The average time intervals from symptom onset to identification, first hospital visit to identification, from symptom onset to the initiation of antiviral therapy with oseltamivir among the patients who died (7.6 ± 4.7 days, 5.5 ± 5.3 days, 7.5 ± 2.4 days, respectively) were significantly longer than those among the patients who survived (4.5 ± 3.7 days, 2.5 ± 3.8 days and 4.5 ± 3.7 days, respectively) (see Supplementary Fig. S1).

A logistic regression analysis indicated that the time intervals from symptom onset to identification and from symptom onset to the initiation of antiviral therapy with oseltamivir greatly affected the number of critical case and the mortality rate of pH1N1 influenza. That is, a delay of 24 h in case identification led to a 9% increase in the odds of patients being classified as critical and a 13% increase in the odds of dying, with odds ratios (ORs) of 1.09 (95%CI: 1.03–1.16, P < 0.01) and 1.13 (95%CI: 1.02–1.26, P < 0.05), respectively, per 24 h increase in the time between symptom onset and identification. A delay of 24 h in initiating antiviral therapy with oseltamivir led to a 12% increase in the odds of patients being classified as critical and an 11% increase in the odds of dying, with ORs of 1.12 (95%CI: 1.03–1.22, P < 0.01) and 1.11 (95% CI: 0.99–1.26, P = 0.086), respectively, per 24 h increase in the time between symptom onset and the initiation of antiviral therapy, though the latter was not significant (Table 2).

### The outcomes of pH1N1 influenza patients with different time intervals between symptom onset and case identification

The basic conditions of the three groups were comparable. However, for the patients identified more than 5 days after symptom onset, the time between symptom onset and hospital admission, the time between onset and the initiation of antiviral therapy, the length of the hospital stay and the course of the disease were significantly longer than those of patients identified within 3–5 days of symptom onset, and these time periods for the patients identified within 3–5 days of symptom onset were also longer than those for patients identified within 2 days of onset. The incidence rates of complications, such as pneumonia, acute respiratory distress syndrome, liver injury and multiple organ failure, were significantly higher among patients identified more than 5 days after symptom onset than those of patients identified within 3–5 days. The rates of the latter group were also significantly higher than those of patients identified within 2 days of symptom onset (Table 3). Eleven (4.7%) of the patients identified more than 5 days after symptom onset, 3 of the patients identified within 3–5 days and 3 (1.2%) of the patients identified within 2 days (1.2%) died. The in-hospital mortality rate of the patients identified more than 5 days later after symptom onset was significantly higher than those of the patients identified within 3–5 days and within 2 days (Table 3).

### The outcomes of pH1N1 influenza patients with different time intervals between symptom onset and the initiation of antiviral therapy

The incidence rate of critical cases, length of the hospital stay, course of disease and the incidence rate of complications such as pneumonia, liver injury and multiple organ failure were also higher among patients who began antiviral therapy more than 5 days after symptom onset than those of patients treated within 3–5 days. These metrics were also higher for the patients treated within 3–5 days than those of patients who received antiviral therapy within 2 days of symptom onset (Table 4). Six (3.4%) of the patients who began antiviral therapy more than 5 days after symptom onset, 4 (1.7%) of the patients who started antiviral therapy within 3–5 days, and none of patients who initiated antiviral therapy within 2 days died. The in-hospital mortality rate among patients initiating antiviral therapy more than 5 days after symptom onset was significantly higher than that of patients initiating antiviral therapy within 3–5 days. The mortality rate for the patients who began antiviral therapy within 3–5 days was also significantly higher than that of patients initiating antiviral therapy within 2 days of symptom onset (Table 4).

### Time intervals between the primary medical activities and the outcomes of pH1N1 influenza patients with PHL-based detection and HCL-based detection

**Figure 1:**
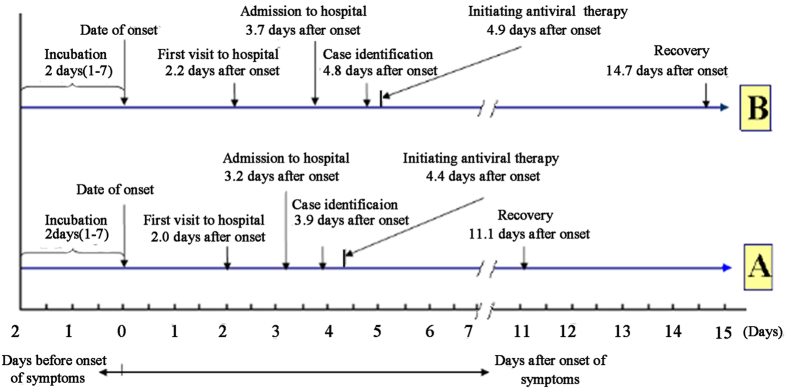
The timetable of major medical activities in the course of infection with pH1N1 influenza. (**A**) Patients in hospitals that could detect the pH1N1 virus. (**B**) Patients in hospitals that could not detect the pH1N1 virus.

The basic conditions of the patients identified by PHLs and HCLs were very similar. However, among patients identified by HCLs, the time intervals between symptom onset and case identification, first hospital visit and case identification, and symptom onset and hospital admission and the lengths of the hospital stay and the course of disease were 3.9 ± 5.0 days, 2.1 ± 2.9 days, 3.2 ± 3.1 days, 7.8 ± 5.7 days and 11.1 ± 7.8 days, respectively; while among patients identified by PHLs, the time intervals between symptom onset and case identification, first hospital visit and case identification, and symptom onset and hospital admission and the length of the hospital stay and the course of disease were 4.8 ± 3.2 days, 3.0 ± 3.4 days, 3.7 ± 3.0 days, 10.2 ± 5.4 days and 14.7 ± 6.8 days, respectively. These lengths of time for patients identified by HCLs were significantly shorter than those for patients by PHLs (P < 0.01, 0.05, 0.01 and 0.01, respectively). The frequency of hospital visits (1.5 ± 0.9 times) among patients identified by HCLs was also significantly lower than that of patients identified by PHLs (1.8 ± 1.2 times) (P < 0.01) (Fig. 1). Due to HCL-based detection, the frequency of hospital visits, the time intervals between symptom onset and case identification, first hospital visit and case identification, and symptom onset to hospital admission and the lengths of the hospital stay and course of disease decreased by 16.7%, 18.8%, 30.0%, 13.5%, 23.5% and 24.5%, respectively (Table 1). Three (1.0%) patients identified by HCLs and 14 (3.2%) patients identified by PHLs died. The in-hospital mortality rate of the former was significantly lower than that of the latter (P < 0.05). With HCL-based detection, the in-hospital mortality rate decreased by 68.8%.

### Detection of the pH1N1 influenza virus in different hospitals

Of the 65 hospitals whose data were available, 29.0% (18) were upper first-class hospitals, 37.1% (23) were middle first-class hospitals, 25.8% (16) were upper second-class hospitals, and 8.1% (5) were middle second-class hospitals. The mortality rates varied in different hospital levels. Of the 65 hospitals, only 13 (20.0%) could conduct the methods required for pH1N1 viral detection, such as RT-PCR and rtRT-PCR detection, during the 2009 pandemic. Among these 13 hospitals, results from 6 needed to be rechecked by the local Center for Disease Control and Prevention. Fifty-two hospitals (80%) could not conduct pH1N1 viral detection, and their specimens were sent to the local Center for Disease Control and Prevention for detection. The reasons why hospitals could not conduct pH1N1 viral detection included prohibition by local authorities and hospitals themselves (80%) and the lack of funds (21.5%), instruments (20.0%) and training (18.5%). Of the 65 hospitals, 40 (61.5%) were less than 10 kilometres from the local Center for Disease Control and Prevention, 15 (23.1%) were within 10–60 kilometres, and 3 (4.6%) were more than 60 kilometres away (see Supplementary Table S2).

## Discussion

Though the H1N1 pandemic has ended, the 2009 pH1N1 influenza outbreak has left us with numerous lessons to summarize and learn. Our findings indicated that a delay of 24 h in case identification led to a 10% increase in the odds of a patient being classified as critical. In severe pH1N1 influenza cases, patients generally begin to deteriorate around 3 to 5 days after symptom onset^10^,^11^. Due to the delayed identification, the incidence rates of complications such as pneumonia, acute respiratory distress syndrome, liver injury and multiple organ failure were significantly increased. A delay of 24 h in identification led to a 13% increase in odds of dying. The average time intervals between symptom onset and identification, first hospital visit and identification, and symptom onset and the initiation of antiviral therapy with oseltamivir among patients who died were longer than those among patients who survived. The in-hospital mortality rate of patients identified more than 5 days after symptom onset was significantly higher than those of patients identified within 3–5 days and within 2 days. A study by Echevarría-Zuno indicated that delayed identification was associated with delayed admission (with an OR of 1.19 per day)^12^. However, our findings indicated that delayed identification not only resulted in delayed admission but also led to delayed antiviral therapy, critical classification and even death.

Many studies assessing the difference in the in-hospital mortality rate between patients given antiviral therapy within 2 days of symptom onset and those started on antiviral therapy 2 days later indicated that the early use of antivirals could also effectively decrease the in-hospital mortality rate^13^,^14^,^15^. In those beyond 2 days of symptom onset who are moderately or severely ill, antivirals could still be beneficial^16^,^17^,^18^. Vernon J. Lee and co-workers provided evidence that early case detection and the use of antiviral ring prophylaxis effectively truncated the spread of infection during an epidemic^19^. Our findings further strengthened the conclusion that delayed antiviral therapy with oseltamivir could increase the in-hospital mortality rate, lengths of the hospital stay and course of disease, and the incidence rates of critical cases and complications, in particular pneumonia, liver injury and multiple organ failure. A delay of 24 h in beginning antiviral therapy with oseltamivir led to a 12% increase in odds of patients being classified as critical. The in-hospital mortality rate among patients who began antiviral therapy more than 5 days after symptom onset was significantly higher than that of patients who began antiviral therapy within 3–5 days. The in-hospital mortality rate of patients who began treatment within 3–5 days was also significantly higher than that of patients initiating antiviral therapy within 2 days of symptom onset. The large number of patients in our study, which enabled us to assess the differences in in-hospital mortality rates among patients who received antiviral therapy within 2 days, within 3–5 days and more than 5 days after symptom onset, indicated that effect of antiviral therapy initiated within 3–5 days was better (or at least significant) than that initiated more than 5 days after symptom onset, though it was not as good as antiviral therapy initiated within 2 days.

Early identification and good patient compliance could improve early antiviral therapy. Our results indicated that a majority of the patients treated with oseltamivir immediately (within approximately 24 h) after being identified exhibited good compliance, but antiviral therapy could be postponed by delayed identification. In fact, the prerequisite for early admission and early antiviral therapy is early case identification. Measures such as developing better diagnostic methods^20^, raising the diagnostic awareness of physicians, and simplifying the workflow of patients visiting hospitals might increase the early identification of patients. The model of HCL-based detection could make detection more convenient and enhance diagnostic abilities and thus facilitate early case identification. Our study indicated that, due to HCL-based detection, the frequency of patients’ hospital visits, the time intervals between symptom onset and case identification, first hospital visit and case identification, and symptom onset to hospital admission, and the lengths of their hospital stay and course of disease decreased by 16.7%, 18.8%, 30.0%, 13.5%, 23.5% and 24.5%, respectively.

According the Law on Prevention and Control of Infectious Diseases^21^, PHLs have an obligation to confirm every suspected case of infectious disease. Some hospitals, considering it a requirement of treatment, are able to detect infectious disease cases. However, during the 2009 pandemic, a low proportion (only 20.0%) of the hospitals were able to perform pH1N1 viral detection. The results from 50% of these hospitals needed to be rechecked by the local Center for Disease Control and Prevention. The main reason for the low proportion of hospitals able to perform pH1N1 viral detection was that it is not supported by hospitals themselves. Though the Ministry of Health of the People’s Republic of China issued a notice on December 7, 2009, that the first-class hospitals were authorized to perform pH1N1 viral detection^22^, local authorities and hospitals preferred to have the detection done by the local CDC laboratories rather than the hospital clinical laboratories in order to ensure biosafety. Another reason for the low proportion was the lack of equipment, funds, staff and techniques. Unlike the detection of bacteria, which has been widely carried out in HCLs^23^,^24^, viral detection was not common in HCLs, especially for emerging viral infectious diseases as pH1N1 influenza^25^,^26^. The WHO Global Influenza Surveillance Network, one of the biggest infectious disease surveillance systems in the detection also obtained its data from PHLs rather than HCLs (the National Influenza Center and sentinel laboratories in each country)^27^,^28^.

Our study indicates that HCL-based detection could improve early identification and early antiviral therapy and thus reduce in-hospital mortality rates. The in-hospital mortality rate among patients with HCL-based detection was significantly lower than that of patients with PHL-based detection. With HCL-based detection, the in-hospital mortality rate decreased by 68.8%. The Department of Infectious Disease in hospitals and community hospitals is the first line of diagnosis and treatment for pH1N1 influenza. Clinical microbiology laboratories could play a key role in the detection and identification of biological agents^29^. Therefore, public health preparedness and health care reform should focus on enhancing the ability of HCLs, including clinical microbiology laboratories and clinical virology laboratories, to detect biological agents. Given that the detection techniques, such as RT-PCR and rtRT-PCR test, are relatively sophisticated, they need to be popularized and enhanced in hospitals^30^. In particular, during the pandemic, the medical and health resources were in relatively short supply, so the HCLs are advised to take on more detection work. Influenza pandemics and epidemics of other infectious diseases are inevitable, so a public health security system should be established to respond to these epidemic or pandemics^31^. A delay in identification and the initiation of antiviral therapy would have a negative effect on the prognosis of patients with pH1N1 influenza, and it could be improved by the model of HCL-based detection. This lesson, though learned from China, could be applicable to other parts of the world in the fight against epidemics or pandemics of influenza.

The measures that should be taken to enhance the ability of HCLs to detect influenza are as follows: first, the local government should increase governmental support, including equipment, special funds and staff; second, hospital administrators should understand the significance and benefits of such detection and support (or at least not prohibit) the involvement of their laboratories; and third, the coordination, communication and collaboration among PHLs and HCLs needs to be further strengthened. By enhancing the ability of HCLs to detect influenza, hospitals would benefit from improved education and collaboration with PHLs, which in turn would result in faster identification of outbreaks and better patient outcomes^32^,^33^.

Undeniably, there were limitations to our study. There was a potential lead-time bias in this study. Severe or critical patients would visit the hospital earlier and draw more attention from physicians. Even if early treatment had no benefit, the time interval between symptom onset and first hospital visit is shorter simply by the addition of the lead time^34^. Second, though the level of hospitals was the same, a hospital with the ability to detect influenza virus, compared to one without this ability, would have more favourable conditions for the treatment of influenza. In addition, though the levels of hospitals the patients with PHL- and HCL-based detection visited were comparable, no specific and detailed methods were taken to assess and compare the treatment capacities of the two groups of hospitals in this study.

## Methods

### The case sources and identification

As pH1N1 became epidemic in China, the Ministry of Public Health of China responded quickly, organizing experts to compile the pH1N1 2009 Clinical Guidelines (1^st^–3^rd^ Edition, 2009)^35^,^36^,^37^, and made an official announcement proclaiming that the diagnosis and treatment of pH1N1 should be conducted in accordance with the guidelines. Hospitalized patients were tracked, and their information was recorded by sentinel investigators. Daily respiratory tract specimens were collected from patients during their hospitalization and tested by real-time RT-PCR to detect the nucleic acid of the 2009 pH1N1 virus. According the Law on Prevention and Control of Infectious Diseases^21^, PHLs are authorized to obtain any suspected infectious disease samples from any hospital. Therefore, the PHLs and HCLs had the same opportunities to obtain samples.

### The diagnosis of cases and classification of cohorts

The confirmed cases of pH1N1 virus infection were defined as any cases confirmed by the Chinese CDC. According to the pH1N1 2009 Clinical Guidelines (1^st^–3^rd^ Edition, 2009) released by the Chinese MOH^35^,^36^,^37^, critical cases were defined as any cases with following symptoms at admission: (1) respiratory failure; (2) septic shock; (3) multiple organs insufficiency; and (4) other critical clinical conditions requiring intensive care. For each patient, we calculated the time between symptom onset and first visit, symptom onset and identification (laboratory confirmed), first hospital visit to identification, symptom onset to the initiation of antiviral therapy, and identification and the initiation of antiviral therapy, as well as length of antiviral therapy time.

A total of 12,894 cases of influenza were reported between May 1 and December 21, 2009, in Zhejiang, China. We planned to randomly investigate 800 cases and were finally able to collect 739 valid cases for our cohort, which accounted for approximately 5.7% of the total number of cases. The cohort was developed by collecting clinical data and laboratory samples. The cohort was divided into two groups according to the different types of laboratories by which their samples were detected: one group was identified by PHLs and the other by HCLs. The cohort was also divided into three subgroups according to the time interval between symptom onset to identification: within 2 days of onset (identification ≤2 days); 3–5 days after onset (identification 3–5 days); more than 5 days after onset (identification >5 days). Antiviral therapy was defined as a therapy with at least 1 day of oseltamivir treatment. The cohort was also divided into three subgroups according to the timeliness of oseltamivir administration: within 2 days after illness onset (oseltamivir ≤2 days); 3–5 days after onset (oseltamivir 3–5 days); more than 5 days after onset (oseltamivir >5 days). We attempted to control for the admission rate bias in this study using a stratification based on the different level of hospitals. Apart from the differences in patients with pH1N1 influenza detected by HCLs or PHLs, the various cases of pH1N1 influenza were randomly distributed to different hospitals, and two groups of patients, separately identified by HCLs and PHLs, were demographically comparable on the presumption that the stratification was based on the different hospital levels.

### Antiviral and symptomatic therapy of pH1N1 influenza

According to the protocol for the diagnosis and treatment of pH1N1 influenza (1^st^–3^rd^ Edition, 2009), antiviral therapy would be used to treat the severe and critical cases and high risk cases infected with influenza A pandemic (H1N1) virus. For adults, oseltamivir was prescribed according to the standard dosing regimen (75 mg twice daily orally for 5 days), and a dosage adjustment would be made for critical cases with a dosing regimen (150 mg twice daily orally for 5 days). For children, a dosage adjustment was made according to their body weight (BW, 30 mg Bid for children with BW < 15 kg; 45 mg Bid for BW 15–23 kg; 60 mg Bid for BW 23–40 kg; and 75 mg Bid for BW > 40 kg).

According to the protocol for the diagnosis and treatment of pH1N1 influenza (1st–3rd Edition, 2009), the appropriate antimicrobial and/or antifungal agents would be administered when patients presented with combined bacterial and/or fungal infections. Patients with hypoxemia or respiratory failure should promptly be given the appropriate oxygen therapy or mechanical ventilation, and patients with combined shock should be given the corresponding anti-shock treatment. In the cohorts, except for oseltamivir, the frequency of administration of other treatments, including antibiotics, traditional Chinese medicine and oxygen therapy, was similar between groups (P > 0.05).

### Data collection

The information was collected in pre-defined standard data collection forms. The investigation, being part of the national database and coordinated by the Chinese Ministry of Health, took place in Zhejiang province, and further information was added about the mode of case identification. The data collection forms were filled in by the hospital sentinels. The sentinel investigators were primarily infectious diseases physicians, closely involved in the care of the patients at their centres. Systematic training was conducted for all sentinel investigators (infectious diseases physicians), including a course covering data collection and methods of detection, diagnosis, treatment and follow-up for pH1N1 influenza. The information collected from the patients was as follows: symptom onset, comorbidities, contact history, date of primary medical onset, visit and admission to hospital(including the frequency of hospital visits: the number of times a patient visited the hospital for outpatient treatment before admission), the types of laboratories by which patients’ samples were detected, antiviral therapy, complications, duration of the viral shedding, prognosis and outcomes. Hospital information, including the hospital class and level, the distance from the hospital to the local CDC, the cooperation between hospitals and the CDC, the ability of the hospital to detect the pH1N1 influenza virus and the reasons why the hospitals could not detect the pH1N1 virus were collected and taken into consideration. The data of collecting main factors for analysis had a low missing rate (less than 7.0%).

### Statistical analysis

The demographic, clinical and laboratory characteristics, time intervals between onset and primary medical activities and outcomes was reported and analysed. Means (standard deviations, SD) or medians (interquartiles, IQR) were calculated as summaries of continuous variables, and incidence numbers (percentage) were calculated as summaries of categorical variables. We compared different demographic and clinical features, time intervals of main medical activities and outcomes by an ANOVA test, chi-square test, or Fisher’s exact test, as appropriate. The risk factor analysis of the independent variables was conducted with a multiple stepwise logistic regression model, in which P = 0.10 was given for the entering level, and P = 0.15 for the excluding level. The testing level was given a = 0.05. The variables used for the multiple logistic regression analysis included the time interval from onset to first visit (days), time interval from onset to identification (days), time interval from onset to the initiation of antiviral therapy (days), time interval from identification to the initiation of antiviral therapy (days) and course of antiviral therapy (days). The data were put into a database in duplicate by different operators, and all the analyses were conducted using SAS version 9.3. Figure 1 was processed by GIMP 2.

### Ethics Statement

The research ethics board at First Affiliated Hospital, School of Medicine, Zhejiang University approved the design and procedure of this study. All methods were performed in accordance with the relevant guidelines and regulations. Informed consent was obtained from all patients.

## Additional Information

**How to cite this article:** Yang, S. *et al*. The need for strengthening the influenza virus detection ability of hospital clinical laboratories: an investigation of the 2009 pandemic. *Sci. Rep.* **7**, 43433; doi: 10.1038/srep43433 (2017).

**Publisher's note:** Springer Nature remains neutral with regard to jurisdictional claims in published maps and institutional affiliations.

## Supplementary Material

Supplementary Information

## Figures and Tables

**Table 1:** Time intervals between the primary medical activities and the outcomes of patients with pH1N1 influenza identified with PHL-based detection and HCL- based detection.

Variables	Patients with PHLs based detection (n = 442)*	Patients with HCLs based detection (n = 297)	Attributable fraction (%)^†^	P value
Base conditions				
Gender-male n (%)	233 (52.7)	174 (58.6)	Null	0.115
Age 0-	110 (24.9)	84 (28.5)	Null	0.460
14-	295 (66.7)	191 (64.7)		
60-	37 (8.4)	20 (6.8)		
BMI < 18.5	108 (27.4)	74 (27.1)	Null	0.316
18.5-	187 (47.5)	146 (53.5)		
25-	68 (17.3)	37 (13.6)		
30-	31 (7.9)	16 (5.9)		
Pulmonary disease n (%)	39 (9.4)	23 (7.9)	Null	0.479
Cardiovascular disease n (%)	55 (13.3)	30 (10.2)	Null	0.221
Metabolic disease n (%)	33 (7.9)	13 (4.4)	Null	0.056
Renal disease n (%)	12 (2.9)	12 (4.1)	Null	0.401
Liver disease n (%)	33 (7.9)	16 (5.4)	Null	0.192
Cancers n (%)	7 (1.7)	9 (3.1)	Null	0.229
Immunosuppression disease n (%)	4 (1.0)	7 (2.4)	Null	0.138
Nervous system disease n (%)	6 (1.4)	7 (2.4)	Null	0.365
Affected elements				
Frequency of visiting to hospital (Times, mean ± SD)^§^	1.8 ± 1.2	1.5 ± 0.9	16.7%	0.004
Time intervals from onset to first visit (Days, mean ± SD)	2.2 ± 3.1	2.0 ± 4.5	9.1%	0.522
Time interval from onset to identification (Days, mean ± SD)	4.8 ± 3.2	3.9 ± 5.0	18.8%	0.003
Time interval from first visit to identification (Days, mean ± SD)	3.0 ± 3.4	2.1 ± 2.9	30.0%	<0.001
Time interval from onset to hospital admission (Days, mean ± SD)	3.7 ± 3.0	3.2 ± 3.1	13.5%	0.045
Time interval from onset to initiating antiviral therapy (Days, mean ± SD)	4.9 ± 3.1	4.4 ± 3.5	10.2%	0.173
Length of stay in hospital (Days, mean ± SD)	10.2 ± 5.4	7.8 ± 5.7	23.5%	<0.001
Course of disease (Days, mean ± SD)	14.7 ± 6.8	11.1 ± 7.8	24.5%	<0.001
In-hospital mortality n (%)	14 (3.2)	3 (1.0)	68.8%	0.043

*The samples were sent to other institutes for detection, such as the local Center for Disease Control and Prevention. ^†^Attributable fraction (%): the proportion of the decreased fraction due to HCL-based detection. Attributable fraction (%) = (values in the group of patients with PHL-based detection - values in the group of patients with HCL-based detection)/values in the group of patients with PHL-based detection. ^§^Frequency of hospital visits (times, mean), which means the number of times a patient visited a hospital for outpatient treatment before admission.

**Table 2:** Multiple logistic regression for analysing the relationship of prognosis with a delay in identification and the initiation of antiviral therapy with oseltamivir.

Variables	Dependent variables	OR (95% CI for OR)*	PAR (%)	P value
Time intervals from onset to first visit (days)	For criticals	1.01 (0.94–1.09)	1.0	0.81
	For deaths	1.00 (0.84–1.17)	0	0.951
Time interval from onset to identification (days)	For criticals	1.09 (1.03–1.16)	9.0	0.005
	For deaths	1.13 (1.02–1.26)	13.0	0.020
Time interval from onset to antiviral therapy(days)	For criticals	1.12 (1.03–1.22)	12.0	0.008
	For deaths	1.11 (0.99–1.26)	11.0	0.086
Time interval from identification to antiviral therapy (days)	For criticals	0.82 (0.41–1.63)	—	0.57
	For deaths	0^‡^	/	0.997
Course of antiviral therapy (days)	For criticals	1.08 (0.97–1.21)	8.0	0.161
	For deaths	1.17 (0.96–1.42)	17.0	0.121

*OR indicates the odds with each per day increase. ^‡^The majority of patients were prescribed antiviral therapy (oseltamivir) immediately after being identified, i.e., within approximately 24 h.

**Table 3:** Time intervals between symptom onset and case identification and the outcomes of patients with pH1N1 influenza.

Variables	Patient identified within 2 days from onset (n = 241)	Patient identified within 3–5 days from onset (257)	Patient identified later than 5 days from onset (234)	P value
Base conditions				
Gender-male n (%)	130 (53.9)	137 (53.3)	134 (57.3)	0.645
Age				
0-	60 (25.1)	74 (28.8)	59 (25.2)	0.876
14-	161 (67.4)	163 (63.4)	156 (66.7)	
60-	18 (7.5)	20 (7.8)	19 (8.1)	
BMI				
<18.5	57 (25.9)	70 (29.8)	55 (26.6)	0.168
18.5-	123 (55.9)	109 (46.4)	98 (47.3)	
25-	24 (10.9)	38 (16.2)	41 (19.8)	
30-	16 (7.3)	18 (7.7)	13 (6.3)	
Pulmonary disease n (%)	22 (9.6)	20 (8.1)	19 (8.5)	0.836
Cardiovascular disease n (%)	21 (9.2)	29 (11.8)	35 (15.5)	0.117
Metabolic disease n (%)	7 (3.1)	19 (7.7)	13 (5.8)	0.077
Renal disease n (%)	9 (3.9)	8 (3.2)	7 (3.1)	0.882
Liver disease n (%)	11 (4.8)	17 (6.9)	23 (10.2)	0.082
Cancers n (%)	5 (2.2)	6 (2.4)	5 (2.2)	0.983
Immunosuppression disease n (%)	3 (1.3)	3 (1.2)	5 (2.2)	0.653
Nervous system disease n (%)	5 (2.2)	4 (1.6)	4 (1.8)	0.892
Pregnancy n (%)	12 (5.2)	14 (5.6)	16 (7.1)	0.679
Complications and Outcomes				
Time interval from onset to hospital admission (Days, mean ± SD)	1.2 ± 1.1	3.2 ± 1.6	6.0 ± 3.6	<0.001
Time interval from onset to initiating antiviral therapy (Days, mean ± SD)	2.1 ± 1.6	4.2 ± 1.4	7.6 ± 3.7	<0.001
Length of stay in hospital (Days, mean ± SD)	9.7 ± 4.8	13.4 ± 6.6	17.6 ± 9.1	<0.001
Course of disease (Days, mean ± SD)	7.5 ± 3.6	9.6 ± 6.0	11.1 ± 6.6	<0.001
Pneumonia n (%)	102 (48.3)	170 (78.7)	166 (86.5)	<0.001
Acute respiratory distress syndrome n (%)	17 (8.1)	24 (11.1)	45 (23.3)	<0.001
Liver injury n (%)	33 (15.6)	34 (15.7)	57 (29.2)	0.001
Renal injury n (%)	9 (4.3)	16 (7.4)	13 (6.7)	0.355
Disseminated intravascular coagulation n (%)	2 (1.0)	1 (0.5)	2 (1.0)	0.760
Septic shock n (%)	2 (0.9)	7 (3.2)	8 (4.1)	0.093
Nervous system complications n (%)	5 (2.4)	8 (3.7)	9 (4.6)	0.456
Multiple organ failure n (%)	5 (2.4)	10 (4.6)	18 (9.2)	0.008
Critical cases n (%)	31 (12.9)	47 (18.3)	64 (27.4)	<0.001
In-hospital mortality n (%)	3 (1.2)	3 (1.2)	11 (4.7)	0.020

**Table 4:** Time intervals between symptom onset and the initiation of antiviral therapy and the outcomes of patients with pH1N1 influenza.

Variables	Antiviral therapy initiating within 2 days from onset (n = 106)	Antiviral therapy initiating in 3–5 days from onset (n = 229)	Antiviral therapy initiating more than 5 days from onset (n = 176)	P value
Base conditions				
Gender-male n (%)	57 (53.8)	111 (48.5)	105 (59.7)	0.082
Age				
0-	29 (27.4)	52 (22.7)	42 (23.9)	0.787
14-	66 (62.3)	159 (69.4)	119 (67.6)	
60-	11 (10.4)	18 (7.9)	15 (8.5)	
BMI				
<18.5	23 (22.8)	55 (26.4)	45 (27.8)	0.690
18.5-	54 (53.5)	106 (51.0)	80 (49.4)	
25-	14 (13.9)	31 (14.9)	29 (17.9)	
30-	10 (9.9)	16 (7.7)	8 (4.9)	
Pulmonary disease n (%)	14 (13.3)	22 (9.6)	14 (8.0)	0.369
Cardiovascular disease n (%)	15 (14.3)	24 (10.6)	30 (17.0)	0.165
Metabolic disease n (%)	7 (6.7)	15 (6.6)	9 (5.1)	0.797
Renal disease n (%)	5 (4.8)	8 (3.5)	6 (3.5)	0.839
Liver disease n (%)	8 (7.6)	17 (7.5)	17 (9.7)	0.697
Cancers n (%)	1 (1.0)	7 (3.1)	4 (2.3)	0.444
Immunosuppression disease n (%)	3 (2.9)	4 (1.8)	3 (1.7)	0.784
Nervous system disease n (%)	2 (1.9)	4 (1.7)	3 (1.7)	0.992
Pregnancy n (%)	6 (5.7)	15 (6.6)	10 (5.7)	0.919
Complication and Outcomes				
Length of stay in hospital (Days, mean ± SD)	8.3 ± 3.7	9.0 ± 5.2	10.9 ± 6.5	0.001
Course of disease (Days, mean ± SD)	11.5 ± 5.1	12.3 ± 5.6	17.0 ± 7.1	<0.001
Pneumonia n (%)	68 (74.7)	152 (74.9)	146 (88.0)	0.003
Acute respiratory distress syndrome n (%)	12 (13.2)	28 (13.7)	33 (20.0)	0.195
Liver injury n (%)	21 (23.1)	30 (14.6)	49 (29.3)	0.002
Renal injury n (%)	5 (5.5)	12 (5.9)	14 (8.4)	0.556
Disseminated intravascular coagulation n (%)	0 (0.0)	1 (0.5)	2 (1.2)	0.385
Septic shock n (%)	4 (4.4)	5 (2.4)	7 (4.2)	0.554
Nervous system complications n (%)	0 (0.0)	7 (3.4)	6 (3.6)	0.055
Multiple organ failure n (%)	3 (3.3)	7 (3.4)	16 (9.6)	0.025
Critical cases n (%)	20 (18.9)	41 (17.9)	49 (27.8)	0.045
In-hospital mortality (crude)	0 (0.0)	4 (1.7)	6 (3.4)	0.043
