# Supplementary material for: The need for strengthening the influenza virus detection ability of hospital clinical laboratories: an investigation of the 2009 pandemic
Source: Sci Rep. 2017 Mar 10;7:43433. doi: 10.1038/srep43433 (PMC5345031; doi:10.1038/srep43433)
Supplement: Supplementary Information [file srep43433-s1.pdf]

The need for strengthening the influenza virus detection ability of hospital clinical laboratories: an investigation of the 2009 pandemic

Shigui Yang[1], Yuqing Zhou[1], Yuanxia Cui[1], Cheng Ding[1], Jie Wu[1], Min Deng[1], Chencheng Wang[1], Xiaoqing Lu[1], Xiaoxiao Chen[1], Yiping Li[2], Dongyan Shi[1], Feng-fang Mi[3], Lanjuan Li[1*]

Author Affiliations:

[1] *State Key Laboratory for Diagnosis and Treatment of Infectious Diseases, Collaborative Innovation Center for Diagnosis and Treatment of Infectious Diseases, The First Affiliated Hospital, College of Medicine, Zhejiang University, Hangzhou 310003, China;*

[2] *Zhejiang Institute of Medical-care Information Technology, Hangzhou 311112, China.*

[3] *Zhejiang Chinese Medical University, Hangzhou 310053, China*

Correspondence author:

Prof. Lanjuan Li, MD.

Email: ljli@zju.edu.cn

Table legends:

Supplementary Table S1:Demographic, symptoms, comorbidities and complications of pH1N1 influenza.* n: the number of patients with a specific symptom or complication; %: percentage of patients with a specific symptom or complication; Duration: the average duration (days) of symptoms or complications with standardized deviation. † Immunosuppressant: patients with HIV/AIDS, or patients who were prescribed immunosuppressant agents, or corticosteroids (equivalent to prednisone 15mg/d, 30 days). ‡ CNS system complications: refer to one or more of the following symptoms: insomnia, restlessness, hallucination, headache, dizziness and abnormal behaviour. ¶ Acute liver damage: AST or ALT > 70 U/L， or Tbil >2mg/dL. § Acute renal failure: Serum Creatinine increased by 2-fold or GFR decreased >50%, or urine<0.5ml/kg/h for at least 12 hours

Supplementary Table S2 : Detection of the pH1N1 influenza virus in different hospitals. * CDC: the center for disease control and prevention.

Supplementary Table S1 Demographic, symptoms, comorbidities and complications of pH1N1 influenza

Variables	Findings (n=739)
**General information**	
Gender-male n (%)	407(55.1)
Age (years) 0-	194(26.3)
14-	486(65.9)
60-	57(7.7)
Body mass index 0-	182(27.3)
18.5	333(49.9)
25.0	105(15.7)
30.0	47(7.0)
Pregnancy	43(6.0)
**Symptoms n(%) / duration(Days,** Mean±SD) *****	
Fever more than 38℃	578(94.5) / 4.8±3.2
Cough	608(96.5) / 10.1±6.1
White sputum	412(65.7) / 8.4±5.2
Nasal congestion	101(16.0) / 4.6±2.6
Sore throat	216(34.6) / 4.9±2.7
Chest distress	209(33.5) / 6.9±6.4
Headache	118(18.9) / 4.4±2.3
Fatigue	206(33.0) / 7.1±4.5
Diarrhea	44(7.0) / 2.8±1.8
**Comorbidities n (%)**	
Respiratory diseases	62(8.8)
Cardiovascular diseases	85(12.0)
Metabolic diseases	39(5.5)
Renal diseases	24(3.4)
Liver diseases	51(7.2)
Cancers	16(2.3)
Immunosuppressant †	11(1.6)
Nervous system disease	13(1.8)
Allergy	58(8.4)
**Complications n(%) / duration(Days,** Mean±SD) *****	
Pneumonia	444(70.9) / 4.5±3.2
Acute respiratory distress syndrome	87(13.9) / 5.6±3.3
Acute liver injury ¶	127(20.2) / 7.1±4.8
Acute renal injury §	38(6.0) / 7.0±7.2
Disseminated intravascular coagulation	5(0.8) / 10.0±5.4
Shock	17(2.7) / 8.2±6.2
CNS system complications ‡	22(3.5) / 5.8±4.5
Multiple organ failure	33(5.2) / 9.1±7.3
**Outcomes n(%)**	
Critical cases	146(19.8%)
In-hospital mortality	17(2.3%)

Supplementary Table S2 Detection of the pH1N1 influenza virus in different hospitals

**Variables**	**Findings(n=65)**
The level of hospitals	
Upper first-class hospitals n (%)	18(29.0)
Middle second-class hospitals n (%)	23(37.1)
Upper second-class hospitals n (%)	16(25.8)
Middle second-class hospitals n (%)	5(8.1)
The distance from hospital to the local CDC*	
0- km n (%)	40(61.5)
10- km n (%)	15(23.1)
>=60 km n (%)	3(4.6)
Hospitals detecting the pH1N1 influenza virus	
Hospitals could detect the pH1N1 influenza virus n (%)	13(20.0)
Using RT-PCR or rtRT-PCR methods n (%)	13(20.0)
Using immunoassay methods n (%)	3(4.6)
Hospitals from which results need to be rechecked by the local CDC n (%)	6(9.2)
Reasons that hospitals couldn't detect the pH1N1 virus	
Detection prohibited by hospital itself or the local government n (%)	52(80.0)
lack of relative personnel n (%)	1(1.5)
No special funds n (%)	14(21.5)
No relative instruments and conditions n (%)	13(20.0)
Lack of relative technique or no special train n (%)	12(18.5)

* CDC: the center for disease control and prevention.

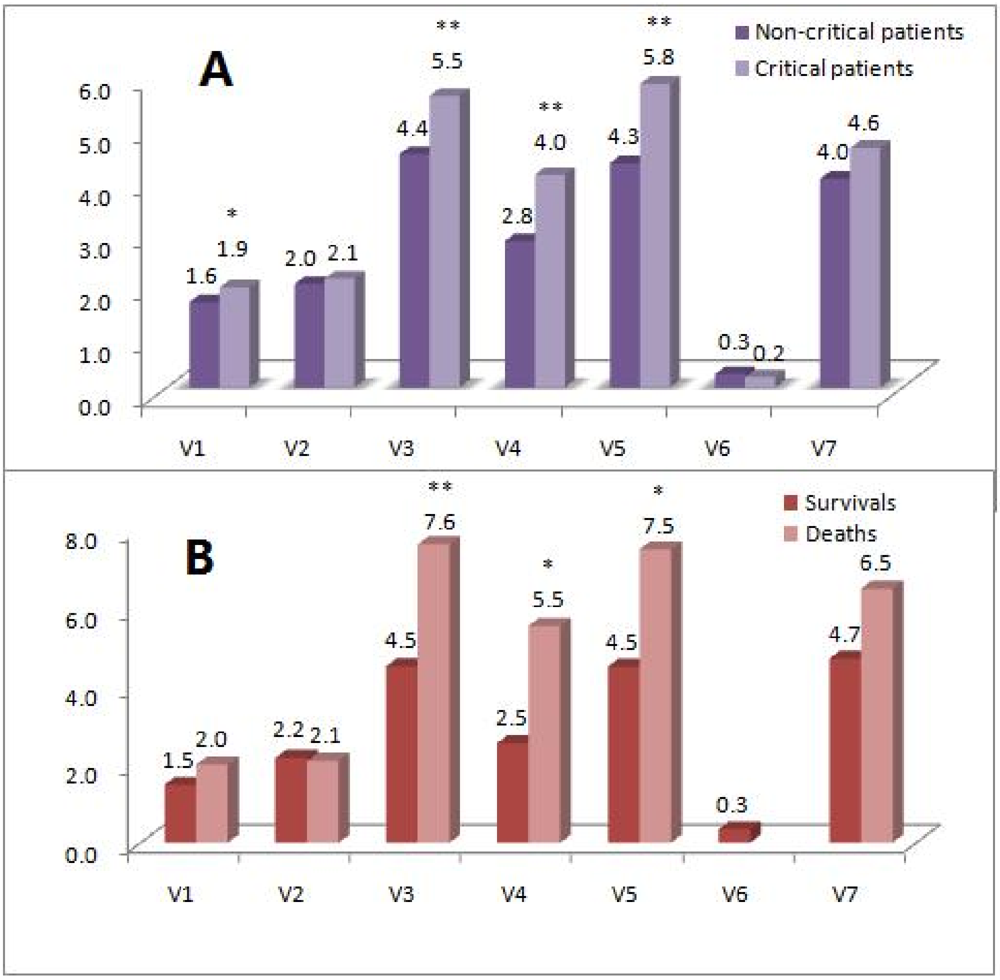

Supplementary Figure S1 Time intervals and frequency of primary medical activities among patients with different outcomes. Legend Panel A: Timeline comparison of the identification and initiation of antiviral therapy with oseltamivir between critical cases and non-critical cases; Panel B: Timeline comparison of the identification and initiation of antiviral therapy with oseltamivir between the patients who survived and the patients who died. V1: Frequency of hospital visits (times, mean), which means the number of times a patient visited a hospital for outpatient treatment before admission; V2: Time intervals between symptom onset and first hospital visit (days, mean); V3: Time interval between symptom onset and identification (days, mean); V4: Time interval between first hospital visit and identification (days, mean); V5: Time interval between symptom onset and the initiation of antiviral therapy (days, mean); V6: Time interval between identification and the initiation of antiviral therapy (days, mean); V7: Course of antiviral therapy (days, mean).
